# Association of Arsenic Exposure with Lung Cancer Incidence Rates in the United States

**DOI:** 10.1371/journal.pone.0025886

**Published:** 2011-10-07

**Authors:** Joseph J. Putila, Nancy Lan Guo

**Affiliations:** 1 Mary Babb Randolph Cancer Center, West Virginia University, Morgantown, West Virginia, United States of America; 2 Department of Community Medicine, West Virginia University, Morgantown, West Virginia, United States of America; Univesity of Texas Southwestern Medical Center at Dallas, United States of America

## Abstract

**Background:**

Although strong exposure to arsenic has been shown to be carcinogenic, its contribution to lung cancer incidence in the United States is not well characterized. We sought to determine if the low-level exposures to arsenic seen in the U.S. are associated with lung cancer incidence after controlling for possible confounders, and to assess the interaction with smoking behavior.

**Methodology:**

Measurements of arsenic stream sediment and soil concentration obtained from the USGS National Geochemical Survey were combined, respectively, with 2008 BRFSS estimates on smoking prevalence and 2000 U.S. Census county level income to determine the effects of these factors on lung cancer incidence, as estimated from respective state-wide cancer registries and the SEER database. Poisson regression was used to determine the association between each variable and age-adjusted county-level lung cancer incidence. ANOVA was used to assess interaction effects between covariates.

**Principal Findings:**

Sediment levels of arsenic were significantly associated with an increase in incident cases of lung cancer (*P*<0.0001). These effects persisted after controlling for smoking and income (*P*<0.0001). Across the U.S., exposure to arsenic may contribute to up to 5,297 lung cancer cases per year. There was also a significant interaction between arsenic exposure levels and smoking prevalence (*P*<0.05).

**Conclusions/Significance:**

Arsenic was significantly associated with lung cancer incidence rates in the U.S. after controlling for smoking and income, indicating that low-level exposure to arsenic is responsible for excess cancer cases in many parts of the U.S. Elevated county smoking prevalence strengthened the association between arsenic exposure and lung cancer incidence rate, an effect previously unseen on a population level.

## Introduction

Arsenic has been linked to multiple cancer types including lung, bladder, kidney, liver, and skin cancers in numerous occupational, epidemiological, and experimental studies [1]–[3]. Studies in Chile, Taiwan, and Bangladesh have established a clear association between arsenic and lung cancer. While some areas of the U.S. have comparable levels, the concentration of arsenic in drinking water is, on the whole, much lower. Smoking is also highly prevalent in many areas of the U.S. and is undoubtedly the leading cause of lung cancer. Tobacco smoke itself also contains arsenic [4]. It is however unclear what effect chronic low-level arsenic exposure has on lung cancer incidence rates in the U.S. independent of and in conjunction with smoking on the population level.

Heavy metal compounds such as arsenic are most commonly introduced into the water supply via naturally occurring deposits, mining, or other disturbances to the soil, which are considered to be one of the main sources of exposure to heavy metals outside of smoking and occupational settings. While some foods may contain low levels of arsenic, the organic forms are not as toxic as the inorganic forms found in the soil and drinking water [5], making exposure via drinking water the primary avenue for reducing population exposure.

Arsenic poses one of the highest cancer risks of any of the common drinking water contaminants, with any given level of arsenic producing 10 to 100 times more cancer cases compared to a similar level of other contaminants [6]. Additionally, there is convincing evidence that there is no biological threshold at which arsenic ceases to be carcinogenic [6], [7], raising the possibility that even low concentrations can have a significant impact on cancer incidence. Exposure to both arsenic and tobacco carcinogens has been seen to confer a greater risk of cancer than exposure to either in isolation in an occupational setting [8]. A synergism between mechanisms of tobacco and heavy metal carcinogenesis could lead to many people being at considerable excess risk even at normally acceptable levels of contamination.

This study sought to determine the relationship between soil arsenic concentrations and lung cancer incidence rates in the U.S., and to elucidate the contribution of this heavy metal to lung cancer rates independent of and in conjunction with smoking. Median county income will be used as a surrogate measure for socioeconomic status, as income and education have been seen to influence incidence rates for lung cancer in other studies using similar data [9].

## Methods

### Patient Data Sources

**Figure 1:**
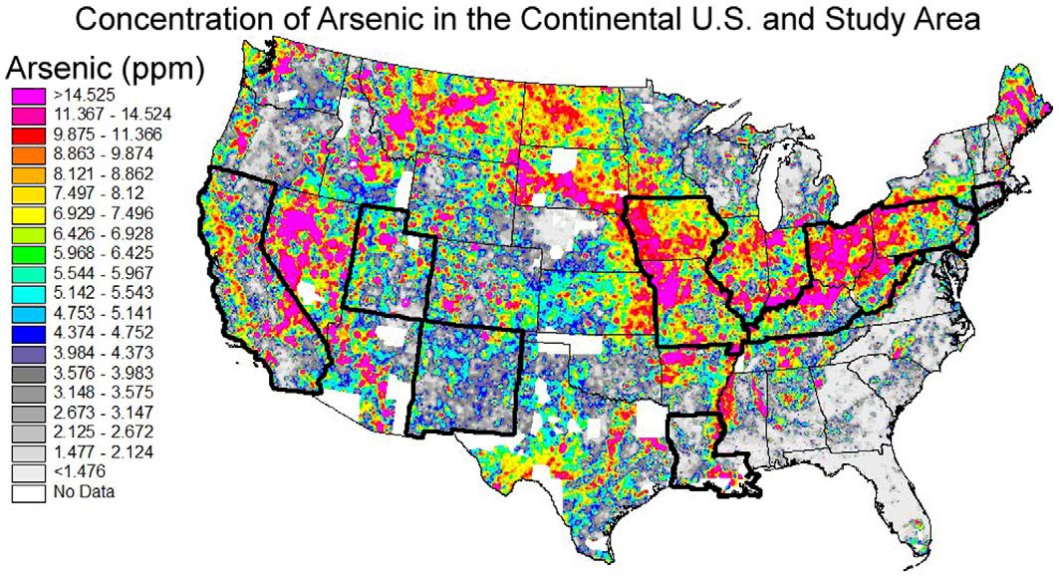
of the extent of coverage and measured levels of arsenic by atomic absorption for the continental U.S. The study area is delineated by a bolded outline. Original image is courtesy of the U.S. Geological Survey.

Individual patient cases for California, Connecticut, Iowa, Kentucky, Louisiana, New Jersey, New Mexico, and Utah were obtained from the Surveillance Epidemiology and End-Results (SEER) database [10]. Criteria for inclusion included the lung or bronchus as the site of the primary tumor, complete data on patient age and county of residence, and a diagnosis between 1996 and 2005. County-level age-adjusted incidence rates for Missouri, Ohio, West Virginia, and Pennsylvania were obtained from the available data published by their respective health departments for a similar time period [11]–[14]. County populations used in age-standardizing rates and weighting regressions, as well as median county incomes were obtained from the 2000 U.S. Census (http://www.census.gov/main/www/cen2000.html). County-level smoking prevalence was calculated using data from the 2008 Behavioral Risk Factor Surveillance System (BRFSS) [15]. Concentrations of arsenic in stream sediments were obtained from the USGS National Geochemical Survey for the 12 states included in the analysis [16]. Individual data elements from separate sources were matched by county and state name or 5-digit FIPS code to form the final data set used in the analyses. Figure 1 shows the study area and the measured levels of arsenic by atomic absorption.

### Age Adjusted Lung Cancer Incidence Rates

Lung cancer incidence rates for counties with data available from SEER were estimated as a ten-year average from 1996–2005 where available, as the sparse population of some counties resulted in unstable single-year estimates. Lung cancer incidence rates calculated from the SEER data were then directly age-adjusted to the 2000 U.S. population using the formula below:
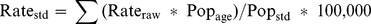
where Rate_std_ is the standardized rate; Rate_raw_ is the un-standardized rate; Pop_age_ is the age-specific population for each age bracket; and Pop_std_ is the total standard population.

Age-adjusted rates of lung cancer were estimated or published for a total of 12 states composed of 757 counties. The states chosen for inclusion represent a demographically, geographically, and geochemically diverse sample of the continental United States. Priority for inclusion was given to the Appalachian region and other areas with high levels of arsenic in order to capture a broad spectrum of exposure.

### Geochemical Data

The mean level of arsenic for each county was calculated by taking the average of all individual point measurements within each county. Multiple analytical techniques were used to measure the levels of arsenic in the original data, including the atomic absorption (AA) [17], Instrumental Neutron Activation Analysis (INAA) [18], and Inductively Coupled Plasma-Atomic Emission Spectrometry 40 (ICP40) [19] methods. These methods offered excellent coverage of the study area, and therefore the data from these three sources were used in the analysis. Arsenic measurements were expressed in terms of parts per million (ppm). The average county exposure level was weighted by its county population in subsequent analyses.

### Smoking Data

County smoking prevalence was calculated as a percent of respondents age 18 or older who reported having smoked more than 100 cigarettes in their lifetime. As the counties with fewer than 30 responses were not available in the survey data, many of the sparsely populated counties were excluded from the models adjusted for smoking.

### Regression Analysis

The first analysis sought to determine the influence of exposure levels of arsenic on lung cancer incidence in the U.S., and the persistence of these effects after controlling for possible confounders. The association between each contaminant and lung cancer incidence was assessed using Poisson regression in order to reflect the annual incidence rate as a counting measure. Poisson regression models the natural logarithm of the outcome, in this case lung cancer incidence rate (Lung_inc_), as a linear function of *n* predictors (*x_1_…x_n_*), each multiplied by a coefficient (*β*) estimated by the maximum likelihood method, plus an error term (e_error_):
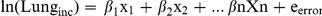



Arsenic was assessed in a model as the single predictor, and in a model controlling for smoking prevalence and median county income expressed as thousands of dollars. Analyses on arsenic levels were able to be weighted by the population of the county to account for varying county size and population density. Regression analyses were performed using the ‘glm’ function with the software package *R* 2.11.0.

### ANOVA Analysis

The second analysis examined two-way interactions between the predictors used in the regression analyses, i.e., heavy metal exposure, smoking, and median county income. The interactions between the variables in the adjusted model were assessed using ANOVA according to the standard formula where the between-group sum of squared error (SS_A_) is divided by the within-group sum of squared error (SS_s/A_).
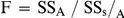



The resulting *F* statistic (*F*), along with the degrees of freedom (DF) then denotes the level of significance of the difference between groups given the variation within groups. With the exception of smoking prevalence which was roughly normal, the covariates were mean-centered to reduce multi-colinearity [20] and log-transformed to better fit a normal distribution before being grouped into quartiles. ANOVA Models used quantized values of arsenic concentration, smoking prevalence, and median income. Lung cancer incidence was untransformed, but the results are presented as log-transformed in the figures in order to simplify the interpretation of the interactions.

### Primary Data and Source Codes

With the exception of the BRFSS and primary SEER data, all of the data and source codes used in the analyses are provided in the online supplement “Files S1, S2, S3, and S4” available in the journal website. The smoking data can be found at the BRFSS website (http://www.cdc.gov/brfss/technical_infodata/surveydata/2008.htm). The aggregated county level lung cancer incidence rates, exposure levels and covariates are included the supplemental files, along with a brief explanation of relevant code.

## Results

### Association between Arsenic and Lung Cancer in the U.S

Poisson regression on the age-adjusted county lung cancer incidence rate showed a significant positive correlation with arsenic levels (*B* = 0.0045, *P*<0.0001) without adjustment for smoking or median county income in the 742 counties with data available. The association between arsenic concentration and lung cancer remained significant after adjusting for smoking rates and median county income (*B* = 0.0039, *P*<0.0001) across 585 counties (Table 1). Smoking was positively and significantly associated with lung cancer incidence in the regression analyses (*P*<0.0001), further confirming that smoking is a strong risk factor of lung cancer incidence in the U.S., even when compared with other risk factors. Median county income was negatively and significantly associated with incidence (*P*<0.0001), indicating that higher income was associated with reduced lung cancer incidence rates in the U.S. Taken together, these results demonstrate that increased exposure to arsenic contributed to higher lung cancer incidence rates in the U.S., independent of smoking and socioeconomic status.

**Table 1:** Summary of Poisson regressions of the effect of arsenic concentration (ppm) on county-level lung cancer incidence rates in the U.S. in an unadjusted model and a model adjusted for both smoking and median county income.

Model and Variable	Coefficient	Std. Error	Odds Ratio (95%CI)	*P*-value	*N*
Unadjusted Model					
*Arsenic*	0.0045	1.9×10^−6^	1.004 (1.004–1.004)	*P*<0.0001	742
Adjusted Model					
*Arsenic*	0.0039	1.9×10^−6^	1.004 (1.004–1.004)	*P*<0.0001	585
*Smoking*	1.80	0.0002	6.061 (6.059–6.064)	*P*<0.0001	585
*Median Income*	−3.54×10^−6^	1.31×10^−9^	0.999 (0.999–0.999)	*P*<0.0001	585

In the joint analysis of arsenic exposure and smoking (Table 1), an increase of 1 ppm of arsenic concentration in the soil was associated with a 0.4% increase in the lung cancer incidence rate (OR: 1.004, 95% CI: [1.004–1.004]). Given a 1% increase in smoking prevalence, the lung cancer incidence rate of the study area increased 6.06 fold (95% CI: [6.059, 6.064]).

For arsenic, the difference in lung cancer incidence rates due to heavy metals between the highest and lowest exposure quantiles is thus 5.3% (Table 2). On a national level, the impact is tremendous. Using an average lung cancer incidence rate of 62.5 cases per 100,000 persons per year, the model coefficients for heavy metal exposure developed on the 12 state samples would indicate that across the U.S. exposure to arsenic may contribute to up to 5,297 lung cancer cases per year when accounting for varying exposure levels.

**Table 2:** Difference in lung cancer incidence attributable to arsenic exposure alone for high and low-exposure areas in the U.S. based on the results of the adjusted Poisson models and USGS survey quantiles in Figure 1.

Compound	Low (ppm)	High (ppm)	β-Estimate	Lung Cancer Rate Increase (%)
*Arsenic*	1.477	14.525	0.0039	5.3%

### ANOVA Analysis of Interactions between Arsenic Exposure, Smoking, and SES

**Figure 2:**
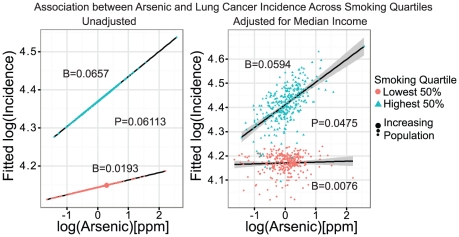
Scatter plot of fitted points showing the estimated association between arsenic and lung cancer incidence in the top 50% (triangles) and lower 50% (circles) smoking quantile. The left figure shows the association without adjustment for median county income, and the right the same association after adjusting for median county income. The size of the dots is proportional to population. Shaded areas delineate the 95% confidence interval for the adjusted model, with the slope of each regression line and *P*-Value for the corresponding ANOVA listed in Table 3.

The ANOVA test for interactions was performed on the mean-centered and log-transformed data. The interaction between arsenic and smoking prevalence was borderline significant (*P* = 0.0611) when comparing quantiles in the model adjusted only for smoking prevalence (Figure 2, left). When adjusted for both smoking prevalence and median income the interaction became significant (*P* = 0.0475; Figure 2 and Table 3). The interaction between arsenic and SES was not significant (*P* = 0.069), and no clear trend was observed from the highest to lowest income quartiles.

**Table 3:** Summary of ANOVA tests performed between Arsenic and covariates used in the regression analysis.

Interaction Pair	DF	*F*-Value	*P*-Value
*Arsenic∶Smoking*	3	2.6595	0.04747
*Arsenic∶MCI*	9	1.7798	0.06914

Figure 2 shows the resulting slopes from a Poisson regression when the data was split into separate quantiles by smoking prevalence for both unadjusted (left) and adjusted (right) models. The increase in the slope of the line from the lower smoking quantile to the higher quantile indicates that as smoking prevalence increases, the association between arsenic exposure and lung cancer incidence strengthens. The P-values on the figures are taken from the respective ANOVA analyses for the adjusted and unadjusted models, with the Beta (*β*) coefficients being taken from the regressions between lung cancer incidence and arsenic exposure specific to a given smoking quantile. Analysis of the interaction between smoking and arsenic concentration showed a clear but insignificant increase in the strength of the association between arsenic and lung cancer incidence with an increase in smoking prevalence (Figure 2, left). This effect became statistically significant after controlling for median income (Figure 2, right). It is also notable that many of the counties with the highest associations are sparsely populated and may represent an already medically underserved population.

### Appalachian Subset

**Figure 3:**
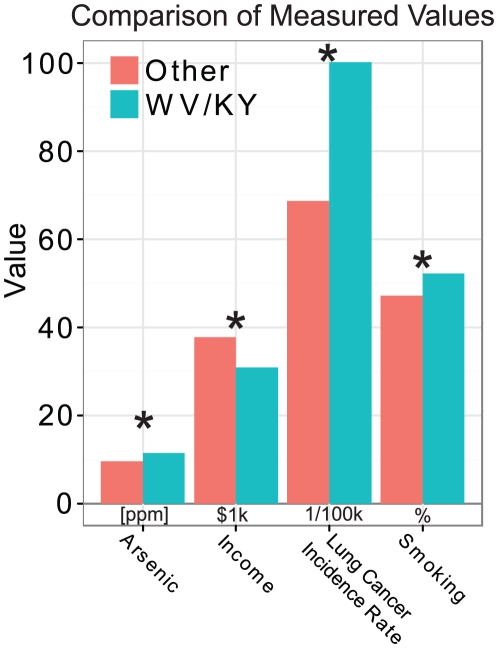
Bar plot showing the average level of exposures and outcomes for counties in West Virginia or Kentucky compared with the remaining 10 states in the original sample. Arsenic exposure is listed as concentration in parts per million (ppm), income as thousands of dollars, incidence rate as annual lung cancer incidence per year per 100,000 persons, and smoking as the percentage of adults having self-reported lifetime smoking. Asterisks (*) indicate statistical significance at *P*<0.05 in the unpaired *t*-test.

**Figure 4:**
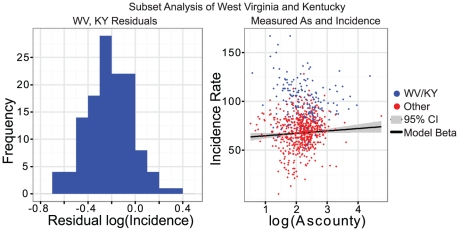
Histogram of the residuals for counties in West Virginia and Kentucky when the model developed on the full 12-state sample was applied (left), with a plot of the measured values for both incidence rate and Arsenic exposure (right). The solid line represents the estimated association between arsenic and lung cancer incidence across the entire sample, with the grey shading representing the 95% confidence interval for the estimate.

West Virginia and Kentucky, two states forming the core of the Appalachian region, have consistently higher lung cancer incidence rates than the national average. In order to further test the relationship between arsenic exposure, smoking, and lung cancer, these two states were compared to counties in the other states on the metrics considered in the regression and ANOVA analyses to determine if the trends seen on the county level in the U.S. held true for these states. A *t*-test of county-level arsenic concentration showed that counties in WV and KY had significantly higher levels of arsenic than did the other counties in the rest of the U.S. under consideration (*P* = 0.015). County-level smoking prevalence for WV and KY was significantly higher than other states considered (*P*<0.0001), while median income was significantly lower (*P*<0.0001). The lung cancer incidence rate was also significantly higher for WV and KY than other states (*P*<0.0001). These results are summarized in Figure 3. A residual analysis showed that the residuals for counties in Kentucky and West Virginia were roughly normally distributed around zero (Figure 4, left), indicating that the model fit with the national data could explain high lung cancer incidence rate seen in WV and KY. Notably, some counties in this subset had comparatively low levels of arsenic but retained a high incidence rate (Figure 4, right); however determining the reason for this is beyond the scope of this analysis. Overall, these results indicate that the higher lung cancer incidence rate in WV and KY is strongly associated with higher arsenic exposure and smoking prevalence as well as lower income in these states. The model presented in this study could be used to explain a portion of the health disparities, specifically higher lung cancer incidence, in the Appalachian region.

## Discussion

This study found that arsenic was a significant predictor of the county-level lung cancer incidence rate in the U.S., and that these effects persisted after controlling for population age composition, smoking prevalence, and median income. There was a clear interaction between smoking behaviors and arsenic contaminant exposure which is observable on the national population level. Areas which had both a higher smoking prevalence and increased arsenic exposure had an excess of lung cancer cases beyond those attributable to either factor, although the presence of other unmeasured factors may attenuate these estimates. This corroborates previous findings in occupational settings which were yet unseen on this scale [8], and is in-line with proposed mechanisms of heavy metal and tobacco carcinogenicity. These findings indicate that the total effect of exposure to arsenic on the population burden of lung cancer may be massively under-estimated due to the pervasiveness of smoking and other tobacco use in the U.S. population.

The results of this analysis indicate that the relationship between arsenic soil concentrations and lung cancer incidence in the U.S. is largely linear and that, barring confounders, a reasonable estimate of the excess incidence due to increased exposure to arsenic can be calculated for a given geographic area. Referring to Figure 1, areas with high levels of arsenic such as those appearing in pink (>14.525 ppm) could attribute a 3.96% increase in the lung cancer incidence rate due to arsenic when compared to an area with a more moderate level such as those counties appearing in blue (4.374–4.752 ppm). Even a more moderate level, such as areas shown in yellow (6.929–7.496 ppm) could attribute a 1.04% increase in lung cancer incidence to arsenic when compared to the same blue areas.

As contamination of the drinking water supply by heavy metals may affect the entire population of a geographic area, the impact of even a small increase in contamination can be quite strong in terms of the total number of excess cases when large populations are affected. For example, Beaver, Greene, and Washington counties along the western edge of Pennsylvania have an elevated level of arsenic, shown as ranging from pink to yellow, when compared to Centre, Mifflin, Perry, and Juniata counties in the center of the state whose arsenic levels range from low (gray) to moderate (blue). The lung cancer incidence rate in the western counties is concordantly higher than those in the central counties (77.4 vs 61.7 per 100,000), a disparity that is likely due at least in part to arsenic exposure and in concordance with the model estimated on the national level. Given the population of the western counties, the elevated arsenic levels may contribute to as many as 28 lung cancer cases per year in that area alone.

There are however a number of limitations to this study. The first is that the design is ecological and these results alone cannot infer an association on the individual level. The second is that the contaminant levels are derived from soil sediment measurements, and the extent to which these contaminants enter into the drinking water supply varies given the amount of soil disturbance, reliance on groundwater, and the type and extent of filtration or purification performed on the water before consumption. The third is that the model does not account for the effect of occupational exposures to arsenic or other carcinogens which have been seen to pose an increased risk of lung cancer. However, as smoking is the most common contributor to lung carcinogenesis and drinking water is one of the most common sources of elevated environmental exposure to heavy metals, it is not expected that occupational exposures to heavy metals or other compounds known to cause lung cancer would bias the results given the size of the study units in relation to the number of occupationally exposed individuals. While vehicle emissions have been shown to be a significant contributor to lung cancer incidence [21], the exposure to both heavy metals and airborne pollutants in concert is not well characterized in terms of its effect on lung cancer incidence.

Because smoking data was not complete for sparsely populated counties which may have higher smoking rates [22], [23], the smoking prevalence used in the adjusted models is likely an under-representation of the true national prevalence. The length of exposure is likely a key factor in the development of cancer. This would likely bias the association between heavy metal exposure and incidence rate towards the null, as persons moving to or from areas of high exposure would be misclassified as to their level of exposure. Future work will consider other important sources of lung cancer incidence such as air quality, such as assessments conducted by the U.S. Environmental Protection Agency (http://www.epa.gov/ttn/atw/nata1999/tables.html), and more refined measures of individual-level smoking behaviors and measurements of heavy metal exposure such as those found in the NHANES data [24].

The findings show that environmental exposure to arsenic may have a significantly larger effect on lung cancer incidence than previously expected in the U.S. Despite the relatively lower concentration of arsenic compared to other areas outside the U.S., it would appear that levels commonly present in the study area still pose a significant risk which is measureable on the population level. In addition, population level smoking prevalence appears to strengthen the effect that arsenic exposure has on lung cancer incidence, resulting in an excess of incident lung cancer cases in areas with high levels of arsenic exposure and smoking. This effect was previously unseen on a large population level in the U.S., and corroborates many of the molecular and occupational studies done on this topic. The observed synergism between arsenic and tobacco use could result in excess risk in individuals with high levels of exposure to both arsenic and tobacco carcinogens.

## Supporting Information

File S1 **The primary exposure data retrieved in 2009 is included in the file “geochem.txt”.**
(TXT)

File S2 **The** ***R*** **source code is included in “source codes.txt”.**
(TXT)

File S3 **Detailed analyses in this study are included in “Documentation_As.doc”.**
(DOC)

File S4 **The aggregated county level lung cancer incidence rates, exposure levels and covariates are included in “county_level_data.csv”.**
(CSV)
