# Supplementary material for: Association of Arsenic Exposure with Lung Cancer Incidence Rates in the United States
Source: PLoS One. 2011 Oct 7;6(10):e25886. doi: 10.1371/journal.pone.0025886 (PMC3189216; doi:10.1371/journal.pone.0025886)
Supplement: File S3 — Detailed analyses in this study are included in “Documentation_As.doc”. (DOC) [file pone.0025886.s003.doc]

Documentation for analysis of Arsenic and Lung Cancer Incidence

The goal of this analysis is to estimate the association between county-level measures of arsenic and county lung cancer incidence rate both in isolation and when controlling for potential confounders, and to assess the interaction effects between covariates. The primary method of analysis for the association of metals and incidence is Poisson regression, and the method of analysis for interactions is ANOVA. A total of 12 states, 8 from SEER and 4 additional, comprised of 757 counties are included.

Data are drawn from multiple independent sources, with each data element being linked to a county by a unique FIPS code made up of 2-digit state and 3-digit county components. The county names are also included as a more intuitive error-checking measure.

The headers and order in this document roughly follow those in the source code.

**Population Data**

The first step is to read in the 2000 Census population data for each county. This data lists the population in each age bracket (0-4, 25-34, 85+, etc) and is used for direct standardization of the estimated rates and as a weight factor in some analyses. The original files, available from <http://www2.census.gov/census_2000/datasets/100_and_sample_profile/> were modified to remove rows representing totals or cities, and columns representing male/female or grand totals.

The files for the 8 SEER states are read individually in .csv format.

*## County-level estimates only from the 2000 U.S. Census*

*CApop <- read.csv("C:/Users/jputila/Desktop/Heavy Metal Data/Census/CA/2kh06_mod.csv")*

A state with an apostrophe (O’Brien) has its apostrophe removed and the files are appended to the Allpop data frame.

*Allpop <- rbind(CApop, CTpop, IApop, KYpop, LApop, NJpop, NMpop, UTpop)*

A shortened name is created and it and the original are moved to the last columns.

*## Shortened name for the county*

*CountyShort <- as.character(substr(Allpop$Area, 1,8))*

*## Move county name to end of data frame*

*Area <- Allpop$Area*

*Allpop <- Allpop[,-1]*

*Allpop <- data.frame(Allpop, CountyShort, Area)*

The 2000 Census data for the entire U.S., in the same format, is read in and subsequently used in rate adjustment.

*## Read population data for the U.S.*

*US <- read.csv("C:/Users/jputila/Desktop/Heavy Metal Data/Census/US/2kh00_mod.csv")*

*## Remove row name, total, males, females columns*

*US <- US[,-c(1:4)]*

A case-listing from SEER is read from all.txt, representing lung cancer cases reported to state registries between 1996 and 2005 (with exception) is used for estimating rates for the SEER states. Entries from California older than 2000 are removed as the entirety of the state was not included until 2000.

*## Import Incidence Data from SEER*

*## IA,KY,LA,NJ,NM,UT,CA,CT*

*Allinc <- read.csv("C:/Users/jputila/Desktop/Heavy Metal Data/SEER/all.txt")*

*## Only 2000+ CA entries*

*Allinc <- Allinc[substr(Allinc$StateCounty,1,2)!="CA" | Allinc$YearDx>=2000,]*

Data from the remaining four states are read in a similar fashion.

*## Missouri, MO - age adjusted*

*MOinc <- read.csv("C:/Users/jputila/Desktop/Heavy Metal Data/Other Inc/MO/mica_mod.csv")*

*MOinc$County <- paste(as.character(factor(MOinc$County)), ", Missouri", sep="")*

*MOinc$FIPSCode <- ifelse(as.numeric(MOinc$FIPSCode) < 10 , paste(0, MOinc$FIPSCode, sep=""), MOinc$FIPSCode)*

*MOinc$FIPSCode <- ifelse(as.numeric(MOinc$FIPSCode) < 100, paste(0, MOinc$FIPSCode, sep=""), MOinc$FIPSCode)*

*MOinc$FIPS <- paste("29", MOinc$FIPSCode, sep="")*

*MOpop <- read.csv("C:/Users/jputila/Desktop/Heavy Metal Data/Other Inc/MO/2kh29_mod.csv", header=TRUE)*

| State | Years Covered | Source |
| --- | --- | --- |
| California | 2000-2005 | SEER |
| Connecticut | 1996-2005 | SEER |
| Iowa | 1996-2005 | SEER |
| Kentucky | 2000-2005 | SEER |
| Louisiana | 2000-2005 | SEER |
| New Jersey | 2000-2005 | SEER |
| New Mexico | 1996-2005 | SEER |
| Utah | 1996-2005 | SEER |
| Missouri | 1998-2006 | [Source](http://health.mo.gov/data/mica/mica/cancer_county_lvl.php) |
| Ohio | 1996-2005 | [Source](http://publicapps.odh.ohio.gov/pwh/PWHMain.aspx?q=072611110817) |
| West Virginia | 2002-2005 | [Source](http://www.wvcancerregistry.org/Portals/27/PDFs/Cancer Incidence in West Virginia 2009.pdf) |
| Pennsylvania | 2003-2005 | [Source](http://app2.health.state.pa.us/epiqms/Asp/SelectParams_Tbl_Cancer.asp) |

Table 1. Summary of sources of cancer incidence and years covered.

Three counties had to be renamed in the incidence file to match the proper format. A FIPS code was then extracted from the registry entries.

*## Create a FIPS code for the SEER data*

*FIPS <- substr(Allinc$StateCounty, nchar(as.character(Allinc$StateCounty))-5, nchar(as.character(Allinc$StateCounty))-1)*

*CountyShort <- substr(Allinc$StateCounty, 5, 12)*

State names were appended to the county names to match the proper format as well.

**Poplation Weights**

Population estimates for each state from the Census were combined by rows and a total population for each county was calculated as the sum of the age-specific populations.

*Pop.1 <- rbind(CApop, CTpop, IApop, KYpop, LApop, NJpop, NMpop, UTpop, MOpop, OHpop, WVpop, PApop)*

*rm(CApop, CTpop, IApop, KYpop, LApop, NJpop, NMpop, UTpop, OHpop, MOpop, WVpop, PApop)*

*## Sum the population of each age group to get the total population*

*Pop <- matrix(NA, nrow=dim(Pop.1)[1], ncol=2)*

*for(i in 1:dim(Pop.1)[1]){*

*Pop[i,1] <- as.character(factor(Pop.1[i,1]))*

*Pop[i,2] <- sum(Pop.1[i,2:14])*

*}*

**Calculate Raw Incidence**

As some of the states in the SEER data have a shorter time-frame, it is necessary to assign the number of years of data available for each county.

*## Years of data available*

*## Either 6 or 10 depending upon when the state entered SEER*

*yearsdata <- NA*

*for(i in 1:NUMCOUNT){*

*yearsdata[i] <- ifelse(unique(substr(Allinc[as.character(Allinc$Area)==as.character(Allpop[i,]$Area),]$StateCounty,1,2)) %in% c("CT", "IA", "NM", "UT"), 10,*

*ifelse(unique(substr(Allinc[as.character(Allinc$Area)==as.character(Allpop[i,]$Area),]$StateCounty,1,2)) %in% c("CA", "LA", "KY", "NJ"), 6, NA))*

*}*

Nested for loops were used to calculate the crude incidence rate for each age bracket (j) in each county (i). These rates are a function of the number of cases in that age bracket and county divided by the age-specific population of that county, expressed as cases per 100,000. A summary of this approach can be found [here](http://www.geo.hunter.cuny.edu/~imiyares/standard.htm).

*for(i in 1:NUMCOUNT){*

*crateRow <- NA*

*for(j in 1:13){*

*crate[i,j] <- ((dim(Allinc[as.character(Allinc$Area)==as.character(Allpop[i,]$Area)*

*& (Allinc$YearDx <= 2005 & Allinc$YearDx >= 1996)*

*& (Allinc$AgeDx >= agroups[j+(j-1)] & Allinc$AgeDx <= agroups[j+j] ),*

*])[1] / yearsdata[i]) / Allpop[i,j])*100000*

*}*

*County[i] <- as.character(Allpop[i,15])*

*FIPS[i] <- as.character(unique(factor(Allinc[as.character(Allinc$Area)==as.character(factor(Allpop[i,]$Area)),]$FIPS)))*

*}*

These crude rates were then adjusted by estimating the sum of the expected cases for each age bracket, and dividing by the total U.S. population.

*## Directly standardizes the rate to the 2000 U.S. population, using the US census data*

*for(i in 1:NUMCOUNT){*

*for(j in 1:13){*

*Allstd[i,j] <- (crate[i,j] * US[1,j])*

*}*

*AdjRate[i] <- (sum(Allstd[i,]) / sum(US[1,]))*

*}*

**SES Data**

SES data, or more specifically Median County Income, was read from individual files available for download from [here](http://www.census.gov/housing/saipe/estmod00/), with documentation available [here](http://www.census.gov/housing/saipe/estmod00/README). The files are read as fixed-width, with the exception of Missouri which appears as a table but is otherwise in the same format. Leading zeros are added to the state and county FIPS codes where necessary.

**BRFSS Data**

Data on smoking was retrieved from the 2008 Behavioral Risk Factor Surveillance System (BRFSS) survey. This data contains information garnered from surveys, with the variables of interest being whether or not the person has smoked 100 cigarettes in their lifetime (SMOKE100) and their county of residence. From this, it is possible to estimate the prevalence of smoking behavior for each county. However, as a means of protecting the survey-takers’ identities the county information is suppressed for counties with a small number of participants. The original data and documentation can be found [here](http://www.cdc.gov/brfss/technical_infodata/surveydata/2008.htm).

The data is read as a fixed width file, with several smoking and demographic variables being pulled.

*wide <- c(2, 95, 1, 1, 1, 2, 29, 3, 1158) #max 1293*

*colname <- c("State", "Junk1", "Smoke100", "SmokeDay", "Quit", "Age", "Junk3", "County", "Junk4")*

*## Read in the data*

*brfss.pre <- read.fwf("C:/Users/jputila/Desktop/Heavy Metal Data/BRFSS/Year08/CDBRFS08.ASC", widths = wide, col.names = colname)*

*## Remove extraneous variables and observations*

*brfss <- brfss.pre[,c(1,3,4,5,6,8)]*

*#rm(brfss.pre)*

*brfss$State <- ifelse(brfss$State < 10, paste("0", brfss$State, sep=""), brfss$State)*

*brfss <- brfss[(brfss$State=="06" |*

*brfss$State=="09" |*

*brfss$State=="19" |*

*brfss$State=="21" |*

*brfss$State=="22" |*

*brfss$State=="29" |*

*brfss$State=="34" |*

*brfss$State=="35" |*

*brfss$State=="39" |*

*brfss$State=="49" |*

*brfss$State=="54" |*

*brfss$State=="42") &*

*!is.na(brfss$State),]*

The sample is restricted to adults…

*brfss <- brfss[brfss$Age>=18,]*

Rates are then calculated as the proportion of the participants from any one county having the appropriate response divided by the total number of participants from that county.

*smk100 <- ifelse(brfss$Smoke100==1, 1, ifelse(brfss$Smoke100==2,0, NA))*

*smkDay <- ifelse(brfss$SmokeDay==1, 1, ifelse(smk100==0 | brfss$SmokeDay==2 | brfss$SmokeDay==3, 0, NA))*

*smkAny <- ifelse(brfss$SmokeDay==1 | brfss$SmokeDay==2, 1, ifelse(brfss$SmokeDay==3 | smk100==0, 0, NA))*

*scfips <- paste(brfss$State, brfss$County, sep="")*

*rateDay <- matrix(rep(NA, NUMCOUNT), NUMCOUNT)*

*rateAny <- matrix(rep(NA, NUMCOUNT), NUMCOUNT)*

*rate100 <- matrix(rep(NA, NUMCOUNT), NUMCOUNT)*

*for(i in 1:NUMCOUNT){*

*rateDay[i] <- (dim(brfss[scfips==as.character(factor(full[i,2])) & smkDay==1 & !is.na(brfss$SmokeDay),])[1] / dim(brfss[scfips==as.character(factor(full[i,2])),])[1])*

*rateAny[i] <- (dim(brfss[scfips==as.character(factor(full[i,2])) & smkAny==1 & !is.na(brfss$SmokeDay),])[1] / dim(brfss[scfips==as.character(factor(full[i,2])),])[1])*

*rate100[i] <- (dim(brfss[scfips==as.character(factor(full[i,2])) & smk100==1 & !is.na(smk100),])[1] / dim(brfss[scfips==as.character(factor(full[i,2])),])[1])*

*}*

**Metal Concentration**

The concentration of Arsenic (ppm) for each county is estimated as the average of all of the measurements within that county. The original data can be downloaded from [here](http://tin.er.usgs.gov/geochem/select.php) for the appropriate counties. The county of origin is determined based on the 5-digit FIPS code.

*## Arsenic*

*## Calculate As values*

*## Do not include "-20" codes in estimates*

*Ascounty <- matrix(NA, nrow = NUMCOUNT, ncol = 1)*

*for(i in 1:NUMCOUNT){*

*Ascounty[i] <- mean(c(na.omit(metal[metal[,42] == as.character(factor(full[i,]$FIPS)) & metal$AS_ICP40 >= 0,]$AS_ICP40) ,*

*na.omit(metal[metal[,42] == as.character(factor(full[i,]$FIPS)) & metal$AS_AA >= 0 ,]$AS_AA),*

*na.omit(metal[metal[,42] == as.character(factor(full[i,]$FIPS)) & metal$AS_INAA >= 0 ,]$AS_INAA)))*

*}*

*Ascounty <- ifelse(Ascounty==0, NA, Ascounty)*

**Statistical Analyses**

The rates are rounded to 0 decimal places to allow for their use in the Poisson models, which typically assess counts.

**Measure Associations**

Log transformed and mean-centered versions of each variable were created for use in the ANOVA analyses, with the exception of the smoking variable which was roughly normal.

Bivariate Poisson regressions were performed, with the concentration of Arsenic being the sole predictor of the incidence rate. The regressions for arsenic were weighted by county population.

*glm1 <- glm(full$AdjRate ~ full$Ascounty, family=poisson, weights=as.numeric(full$Population))*

Adjusted models were also estimated, controlling for smoking prevalence (smkrate) and median income (MedIncome),with the arsenic regressions again being weighted.

*SESassmk <- glm(full$AdjRate ~ full$smkrate + full$Ascounty + full$MedIncome, family=poisson, weights=as.numeric(full$Population))*

The next step was to estimate the 25%, 50% (median), and 75% quartile points for the ANOVA models. This was accomplished by extracting this information from as elements from the ‘summary’ function.

*AsCut <- NA*

*AsCut[1] <- as.numeric(summary(full$lnAs)[2])*

*AsCut[2] <- as.numeric(summary(full$lnAs)[3])*

*AsCut[3] <- as.numeric(summary(full$lnAs)[5])*

Continuous interaction models were estimated by multiplying the mean-centered and log-transformed variables in question with each other and entering that new value into an ANOVA model with the other covariates.

*intAsSmk <- aov(full$AdjRate ~ SESgrpf + AsQf*smkgrpf, weights=as.numeric(full$Population))*

For the quartile-based interaction models, each county was assigned a value from 1-4 to represent its quartile in each variable.

*smkgrp <- ifelse(is.na(full$lnsmk), NA, ifelse(full$lnsmk < SmkCut[1], 1, ifelse(full$lnsmk >= SmkCut[1] & full$lnsmk < SmkCut[2], 2, ifelse(full$lnsmk >= SmkCut[2] & full$lnsmk < SmkCut[3], 3, 4))))*

These were then converted to factors and used for each interaction model.

*intAsSmk <- aov(full$AdjRate ~ SESgrpf + AsQf*smkgrpf, weights=as.numeric(full$Population))*

The interactions were evaluated for both smoking and median income with arsenic concentration. A new regression was estimated using only counties from a specific quartile, and their slopes plotted in order to assess the effect of smoking or income on the relationship between exposure and incidence. In some cases, quartiles were combined before plotting. Plotting was done using the ‘ggplot’ package, with bands representing 95% confidence intervals.

**West Virginia / Kentucky Analyses**

The next step was to analyze the model developed on the 12 states using only data from West Virginia and Kentucky. Three arsenic models, a bivariate and two adjusted models were estimated to be used in subsequent analyses.

*## Bivariate, National*

*glm1 <- glm(full$AdjRate ~ full$lnAs, family=poisson, weights=as.numeric(full$Population))*

*summary(glm1)*

*plot(full$AdjRate, full$lnAs, xlab="Cancer Incidence per 100,000", ylab="As [ppm]")*

*abline(a=glm1$coef[1], b=glm1$coef[2], col=1)*

*## Adjusted for Smoking, National*

*glm2 <- glm(full$AdjRate ~ full$smkrate + full$Ascounty, family=poisson, weights=as.numeric(full$Population))*

*summary(glm2)*

*## Adjusted for Smoking, Income, National*

*glm3 <- glm(full$AdjRate ~ full$smkrate + full$Ascounty + full$MedIncome, family=poisson, weights=as.numeric(full$Population))*

*summary(glm3)*

The ‘predict’ function was then used to estimate incidence for WV/KY based on the national model.

*pred1 <- predict(glm3, full)*

The difference between the predicted and actual incidence was calculated and a histogram of the residuals for WV/KY and plot of actual data was drawn.

*## GGplot*

*full$wvkyid <- ifelse(full$SFIPS==54 | full$SFIPS==21, 1, 0)*

*cols <- c("0" = "red","1" = "blue")*

*p1 <- ggplot(full, aes(y=AdjRate, x=log(Ascounty), weight=Population, colour=full$wvkyid))*

*p1 + stat_smooth(method=glm, level=0.95, alpha=1, fill="grey80", color="black") + geom_point() + scale_colour_manual(values = cols, limits = c("0", "1")) + theme_bw()*

*## Compare residuals for predicting on WVKY using the National fully controlled model*

*pred1 <- predict(glm3, full)*

*hist(pred1)*

*diffresid <- pred1 - log(full$AdjRate)*

*resids <- data.frame(as.character(full$County), pred1, full$AdjRate, diffresid)*

*qplot(diffresid[full$wvkyid==1], data=resids, geom="histogram") + theme_bw()*

Lastly, the average for both WV/KY and other counties was calculated for all of the variables. A t-test was used to determine if there was a significant difference between counties in WV/KY and counties in other states for each variable.

## Arsenic

KYWVas <- mean(na.omit(full[full$SFIPS==21 | full$SFIPS==54,]$Ascounty))

KYWVas

notKYWVas <- mean(na.omit(full[full$SFIPS!=21 & full$SFIPS!=54,]$Ascounty))

notKYWVas

t.test(na.omit(full[full$SFIPS==21 | full$SFIPS==54,]$Ascounty),

na.omit(full[full$SFIPS!=21 & full$SFIPS!=54,]$Ascounty))

The averages were then drawn in a bar chart.

*## Draw a bar chart of the values used in the T-tests*

*bardata <- c(notKYWVas, KYWVas,*

*notKYWVsmk*100, KYWVsmk*100, notKYWVmed/1000, KYWVmed/1000, notKYWVrate, KYWVrate)*

*barcats <- c("Arsenic", "Arsenic",*

*"Smoking", "Smoking", "Income", "Income", "Rate", "Rate")*

*barid <- c("Other", "WV/KY",*

*"Other", "WV/KY", "Other", "WV/KY", "Other", "WV/KY")*

*bardata1 <- data.frame(barid, barcats, bardata)*

*names(bardata1) <- c("ID", "Category", "Values")*

*bar1 <- ggplot(bardata1, aes(Category, fill=ID, y=Values))*

*bar1 + geom_bar(position="dodge", stat="identity") +*

*theme_bw()*

[END]
